# Analysis of the APOB Gene and Apolipoprotein B Serum Levels in a Mexican Population with Acute Coronary Syndrome: Association with the Single Nucleotide Variants rs1469513, rs673548, rs676210, and rs1042034

**DOI:** 10.1155/2022/4901090

**Published:** 2022-03-31

**Authors:** Maricela Aceves-Ramírez, Yeminia Valle, Fidel Casillas-Muñoz, Diana Emilia Martínez-Fernández, Brenda Parra-Reyna, Víctor Arturo López-Moreno, Héctor Enrique Flores-Salinas, Emmanuel Valdés-Alvarado, José Francisco Muñoz-Valle, Texali García-Garduño, Jorge Ramón Padilla-Gutiérrez

**Affiliations:** ^1^Instituto de Investigación en Ciencias Biomédicas (IICB), Centro Universitario de Ciencias de La Salud (CUCS), Universidad de Guadalajara (UDG), Guadalajara, Jalisco, Mexico; ^2^Doctorado en Genética Humana (DGH), Centro Universitario de Ciencias de La Salud (CUCS), Universidad de Guadalajara (UDG), Guadalajara, Jalisco, Mexico; ^3^Especialidad en Cardiología, Unidad Médica de Alta Especialidad, Centro Médico Nacional de Occidente (CMNO), Departamento de Cardiología, Instituto Mexicano Del Seguro Social (IMSS), Guadalajara, Jalisco, Mexico

## Abstract

Apolipoprotein B (APOB) is associated with the development of atherosclerosis and consequently in the acute coronary syndrome (ACS) physiopathology. Single number variants (SNVs) in apolipoprotein B gene (APOB) influence over the susceptibility for this syndrome. The aim of this study was to determine the impact of the rs1469513, rs673548, rs676210, and rs1042034 SNVs and serum levels of APOB in the risk of ACS in a population from western Mexico. We included 300 patients in the group of cases (ACSG) and 300 individuals in the control group (CG). APOB levels were evaluated by immunonephelometry, and SNVs were genotyped with TaqMan probes. We found significant allelic and genotypic differences between groups for rs673548 and rs676210 (OR = 1.33, *p*=0.030, OR = 2.69, *p* < 0.001) and rs1042034 (OR = 0.50, *p*=0.037) SNVs. We found a risk haplotype TAGT (OR: 2.14, IC 1.50–3.04, *p* < 0.001). Our findings support a significant risk association between rs673548 and rs676210 variants for ACS; meanwhile, rs1042034 could be considered protective factor in a western Mexican population. Also, in this population, haplotype TAGT may confer 2.14 times a higher risk. APOB serum levels were compared by genotype variants in both groups without any significant statistical difference.

## 1. Introduction

In the world, cardiovascular diseases (CVDs) represent the first cause of death [1]. Acute coronary syndrome, a part of CVD, comprises unstable angina (UA), non-ST-segment elevation myocardial infarction (NSTEMI), and ST-segment elevation acute myocardial infarction (STEMI). These diseases differ in the degree of severity and are characterized by myocardial ischemia [2], caused by partial or total obstruction of the coronary circulation due to the disruption of an atherosclerotic plaque [3].

Apolipoprotein B (APOB) is a glycoprotein that participates in the assembly and secretion of lipids and in the intravascular transport and delivery of distinct lipoproteins related to the atherosclerotic plaque formation, principally low-density lipoproteins (LDLs) [4, 5].

Trapping of the LDL within the arterial wall is the step that initiates and drives to the complications of the atherosclerotic [4, 6].

APOB is encoded by the *APOB*, a gene with 29 exons and 28 introns in its structure, located in chromosome 2 [7], and many SNVs in this gene have been associated with disorders in lipid metabolism, a main risk factor observed in patients with atherosclerosis [8].

SNV rs676210 has been reported as a variant associated with low-density lipoprotein (LDL) oxidation, and it should be noted that this is a major risk factor in the pathophysiology of atherosclerosis. [9]. In Chinese population, rs676210 and rs1042034 SNVs have been associated with myocardial infarction as a cause of hyperlipidemia and are a factor for having higher levels of APOB [10, 11]. Also, SNV rs1469513 has been reported to be associated with significant differences between carriers of allele G vs. A for total cholesterol and LDL levels and with other ACS risk factors like overweight and obesity in a Korean population [12]. Variant rs673548 has been associated with other diseases related to disturbances in lipid metabolism [13] and ischemia [14], but its involvement in ACS has not been explored.

In Mexico, heart disease is the number one cause of death [15]; for this reason, the objective of this study was to determine the impact of the rs1469513, rs673548, rs676210, and rs1042034 *APOB* gene variants and APOB levels of in the risk of acute coronary syndrome in a Mexican population of western Mexico.

## 2. Materials and Methods

### 2.1. Ethical Compliance

The study was performed according to the ethical principles for experiments involving humans stated on the Declaration of Helsinki, and ethical approval was obtained by the Centro Universitario de Ciencias de la Salud, CUCS, UdeG (C.I. 065–2014). Informed consent was obtained from all patients for being included in the study.

### 2.2. Study Population

Six hundred unrelated individuals were included in this study, 300 in the case group (ACSG) and 300 in the control group (CG). In the ACS group, the inclusion criteria included diagnosis according to the guidelines of the American College of Cardiology [16]. As a control group, the inclusion criteria included individuals with a similar age to the cases. As exclusion criteria in ACS, we excluded individuals with familial hypercholesterolemia, individuals with overlapping other cardiac or noncardiac diseases (e.g., pulmonary embolism, pulmonary infarction, pneumothorax, pleurisy, pneumonia, anemia, aortic dissection, aortic aneurysm, esophageal spasm, cerebrovascular disease), and genetically related individuals. In CG, we exclude individuals with a personal history of ischemic heart disease and familial hypercholesterolemia, and genetically related individual. In both groups, we exclude individuals who received a blood transfusion in the last three months before sampling.

In the ACS group, a review of the clinical record was carried out, from which data such as blood pressure, troponin levels, creatine kinase (CK), and creatine kinase MB (CK-MB) were obtained. All clinical data on height, weight, comorbidities, and lifestyle were obtained by questionnaire in both groups. The individuals in both groups are from western Mexico, as are their parents and grandparents.

### 2.3. Genetic Analysis

Genotyping was carried out by allelic discrimination using TaqMan probes (rs1469513, rs673548, rs676210, rs1042034) (catalog 4351379) (Applied Biosystems, Foster City, CA, USA) according to the manufacturer's instructions. As a quality control, 25% of the samples were double genotyped and a 100% matching was obtained.

### 2.4. Quantification of Serum APOB Levels

APOB levels were measured by using a turbidimetry technique [17] according to the manufacturer's instructions (BioSystems) on a Mindray BS-120 equipment from Mindray Medical España S.L.

### 2.5. Biochemical Profile

The C-reactive protein (CRP) was quantified using the latex technique [18], and the quantification of cholesterol [19, 20], high-density lipoprotein (HDL) [21], LDL [22], triglycerides [23, 24], and glucose [25] was carried out using enzymatic methods according to the indications of BioSystems S.A.

### 2.6. Analysis of Linkage Disequilibrium (LD)

Haplotypes were inferred by the expectation maximization algorithm in the order rs1469513, rs673548, rs676210, and rs1042034. The normalized LD (D′) and squared correlation of allele frequencies (*r*^2^) were estimated using the available online SHEsis program [26]. We exclude haplotypes with a frequency less than 5%.

### 2.7. Statistical Analysis

Statistical analysis was performed using SPSS® version 22.0 and Excel® 2010. The data for continuous variables are presented as the mean and interquartile range, and differences between groups were compared with the Mann–Whitney U test. The *χ*2 test or Fisher's exact test was used to compare discrete variables and to estimate the Hardy–Weinberg equilibrium. Allele and genotype frequencies were obtained by direct counting. Dominant and recessive allele models were tested with *χ*2 test for each SNV. Under the dominant model, the heterozygous and homozygous genotypes of the minor allele were compared with the homozygous genotype of the major allele and, under the recessive model, were compared the heterozygous and homozygous genotypes of the major allele with the homozygous genotype of the minor allele. We performed a bivariate logistic regression where the dependent variable was the genetic models; however, the omnibus test was not significant (data not shown).

We performed a linear regression model to adjust the APOB serum levels by independent variables.

The odds ratio (OR) was the measure of association for genotype and allele frequencies and for risk factors with a confidence interval of 95%. The cutoff of significance was *p* < 0.05.

## 3. Results

### 3.1. Clinical and Demographic Characteristics

The mean age of individuals in ACSG and in individuals in CG was 64.25 and 57.76 years, respectively. In the group of cases, the frequency of ACS was 3.2 times higher in men than in women. STEMI was the most prevalent type of ACS (70%). Reinfarction was the reason for hospitalization in 17% of cases. High blood pressure was the main prevalent risk factor in both groups; however, risk factors are overrepresented in the ACS group (Table 1).

### 3.2. Biochemical Parameters

Except for glucose (125.4 mg/dL in CG and 144.15 mg/dL in ACSG), cholesterol, triglycerides, HDL, and LDL levels were normal in both groups (Table 2).

### 3.3. Genotype and Allele Distribution

The genetic variants studied rs1469513, rs673548, rs676210, and rs1042034 were found in Hardy–Weinberg equilibrium, *p* ≥ 0.13. No significant differences were found between groups for the allelic and genotype distributions of the rs1469513 variant. The rs673548 showed a risk contribution, in which the G/A genotype presented an OR of 1.53 (*p*=0.014), the A allele an OR of 1.33 (*p*=0.030), and the dominant model an OR of 1.50 (*p*=0.013). Carriers of genotype A/A of the rs676210 showed and increased risk (OR = 2.69, *p* < 0.001); furthermore, it was found that allele A confers a higher risk compared to allele G (OR = 1.72, *p* < 0.001) (Table 3), which is confirmed by the analysis under the dominant model (OR = 1.81, *p* < 0.001) (Table 4). Genotype T/T of the rs1042034 showed a protective contribution (OR = 0.50, *p*=0.037) (Table 3), and this was confirmed by the recessive inheritance model, through which we found that the presence of two T alleles confers protection (OR = 0.49, *p*=0.028) (Table 4).

### 3.4. Analysis of Linkage Disequilibrium

We found linkage disequilibrium between the pair variants in the control group, rs1469513-rs673548 D′ = 0.69 (*p* < 0.0001), *r*^2^ = 0.11 (*p* < 0.0001), rs1469513-rs676210 *D*′ = 0.83 (*p* < 0.0001), *r*^2^ = 0.15 (*p* < 0.0001), rs1469513-rs1042034 *D*′ = 0.37 (*p* < 0.0001), *r*^2^ = 0.03 (*p* < 0.0001), rs673548-rs676210 *D*′ = 0.80 (*p* < 0.0001), *r*^2^ = 0.60 (*p* < 0.0001), rs673548-rs1042034 *D*′ = 0.53 (*p* < 0.0001), *r*^2^ = 0.26 (*p* < 0.0001), rs676210-rs1042034 *D*′ = 0.49 (*p* < 0.0001), *r*^2^ = 0.21 (*p* < 0.0001), and compared the haplotype distribution between groups, and we found that the haplotype TAGT confers a risk 2.14 times higher (*p* < 0.001) (Table 5).

### 3.5. APOB Serum Levels

APOB serum levels were found in the normal range in both groups (Table 2); however, under the recessive model in CG, a statistical difference was found in the rs1469513 (T/T + T/C:179 vs. C/C:149.7 mg/dl, *p*=0.0001).

### 3.6. The Dependent Variable

APOB serum levels were adjusted by independent variables age, sex, comorbidities, treatment, and biochemical parameters (cholesterol, triglycerides, glucose, HDL, LDL), and cholesterol levels (*β* = 0.779, CI = 0.633–0.926, *p* ≤ 0.001) and HDL (*β* = −0.597, CI = −0.935–0.258, *p*=0.001) showed a significative association with APOB serum levels (Table 6).

## 4. Discussion

In our study, we found that in the ACS group, the average age was 64.25 years and that the male sex was predominant (76%); furthermore, high blood pressure was the main risk factor, being present in 70% of the individuals in the ACS group, and similar results have been found regarding RENASCA (National Registry of the Acute Coronary Syndrome) report, in which 75% of male were reported. In this cohort, the main risk factor was high blood pressure, present in 60.5% of individuals [27].

In the ACCESS study, which includes individuals from Mexico, it was found that 72% of the patients were men, according to our findings, and the most frequent risk factor was high blood pressure (62% of participants), but the average age of presentation was 62.1 years [28]. The foregoing highlights age, sex, and other main risk factors associated with ACS.

STEMI, the severe entity of ACS, was the most frequent in our study, in contrast to the ACCESS study where the most frequent entities were UA and NSTEMI, which together made up 51.9% of cases compared to a 40.3% of cases in our study. The average age of presentation was 62.1 years, similar to our findings, where the average was 57.76 years in females and 64.25 in males [28].

In the control group, we find higher percentages of individuals with diabetes (22.7%), high blood pressure (34.7%), dyslipidemia (20.3%), and smoking (20.7%); however, the percentages of overweight (23.7%), obesity (16.3%), and sedentary lifestyle (23%) were lower compared to what was reported in the National Health and Nutrition Survey (ENSANUT) carried out in 2018, where 8.6 million (10.3%) have a diagnosis of diabetes mellitus and 15.2 million (18.4%) a diagnosis of high blood pressure, registering an increase in the incidence from 50 years of age; therefore, in the age group 70 to 79 years, the proportion of individuals with this disease reaches 26.7%. The survey also reports 19.5% of individuals with elevated triglyceride and cholesterol levels, obesity in 36.1%, and overweight in 39.1%. Regarding physical activity, 29% of individuals in this age group are sedentary. It was found that tobacco consumption in Jalisco represents 12.6%, in Colima 12.3%, the Michoacán 12.7%, and in Nayarit 9.8%, and our study subjects come from these regions of the country [29].

Compared to the ACS group, in CG, we found higher levels of cholesterol, triglycerides, HDL, LDL, and APOB, a difference not seen in glucose or C-reactive protein. All other parameters were within normal range; 89% of individuals in the case group take antilipemic drugs regularly, which is a factor that favors the reduction of lipid levels in patients with ACS. Among the antilipemic drugs that are consumed are statins (reduce cholesterol levels) [30] and bezafibrate (reduce triglyceride levels) [31]. Statins affect LDL and APOB levels, so it is possible to find lower levels of these parameters in our ACS group. Statins also decrease the number of circulating APOB particles by decreasing VLDL synthesis and thus the production of VLDL and LDL remnants; furthermore, they increase the clearance of these particles through the upregulation of LDL receptors in the liver [32].

Medication to lower lipid levels in the ACS group denotes that lipid control is not a factor that by itself prevents the occurrence of an ACS event, but that there will be other genetic and environmental risk factors that are causing the occurrence of this event; since we found individuals in CG with higher levels of blood lipids, despite being of similar age to individuals in the ACS group, they have not presented an ACS event.

In addition, it has been shown that lipid levels can be modified after an event of ACS, with a trend toward statistically significant decreases in total cholesterol, low-density lipid cholesterol and high-density lipid cholesterol, and a trend toward increased triglyceride levels [33].

We found significative lower levels of HDL in the ACS group compared with CG (28.4 mg/dL vs. 41.6 mg/dL, *p* < 0.001). Low HDL levels have been associated with ACS for its antiatherogenic properties, and Cordero et al. found significantly lower levels in the ACS group (*p* < 0.01) of HDL (average 36.1 mg/dL) compared to patients with nonischemic chest pain (average, 44.5 mg/dL). An increase of 1 mg/dL in the levels of this lipoprotein has been found to decrease the risk of coronary heart disease by 2‐3% [34].

We documented a higher proportion of sedentary individuals in ACS; a known factor in reducing contributes to HDL levels. The impact of physical activity has been evidenced by Skoumas et al, and they found that physically active individuals have higher levels of HDL compared to sedentary (*p* < 0.5) [35].

C-Reactive protein is a serum marker to assess cardiovascular risk. Healthy people have 2–4 times higher levels, which have been associated with increased risk; furthermore, it is an acute phase reactant that rises in the event of an infection or tissue damage [36]. Given this situation, in ACSG, we found considerably higher levels (26.18 mg/L). In CG, the mean C-reactive protein serum level was within normal range (4.2 mg/L).

Acute coronary syndrome is of multifactorial origin, and among the associated factors is APOB since it is considered a predictor for its occurrence [37]. Variants in the *APOB* gene have been associated as protective or risk factors for ACS [38–40].

We included in the study two missense variants, rs676210 (NP_000375.3: p. Pro2739Leu) [41] and rs1042034 (NP_000375.3: p. Ser4338Asn) [41], and the intronic variants rs1469513 and rs673548. In the present investigation, no risk nor protection association was found with the rs1469513 (T > C) variant; however, in the Korean population, the lower allele has been associated with a higher risk of obesity (OR = 1.31, *p*=0.004) and higher levels of total cholesterol (*p*=0.001) and LDL (*p*=0.01) [14]. According to the analysis performed in regSNP-intron, this variant does not affect the splicing site [42].

We found a risk association with genotype G/A in the rs673548 (OR = 1.53, *p*=0.014), and under the dominant model, we found that only one copy of allele A is necessary for an incremented risk (OR = 1.50, *p*=0.013). The risk has not been previously evaluated in ACS, although it has been related to ischemia, in ischemic stroke where G allele increases the risk 1.28 times (*p*=0.034) [14]. According to the results obtained in regSNP-intron, this variant does not affect the splicing site [42].

The rs676210 variant appears to be an important risk factor in other populations as well as in our study. We found a risk association for A allele (OR 2.69, *p* < 0.001). Mäkelä et al. found that this variant is associated with LDL oxidation, a factor associated with atherosclerosis, but in contrast with our results, allele G confers 1.28 times higher risk of coronary disease (*p*=0.030). In addition, A allele in control subjects has shown higher serum lipid levels (*p*=0.01) [9]. In contrast to these findings, in the Chinese population, it was found that carriers of the G/G genotype present an increased risk of ACS (OR = 1.93, *p*=0.005 compared to carriers of the A/A genotype) [11]. Variants of rs676210 (*p*=0.005) and rs1042034 (*p*=0.009) have been found as a risk factor in Chinese population for hyperlipidemia, an important risk factor for atherosclerosis and coronary artery disease [10]. According to the report obtained from the analysis *in silico* in the HOPE database, the variant, rs676210 (P2739L), the mutant residue is bigger than the wild-type residue. Prolines are known to be very rigid and therefore induce a special backbone conformation, which might be required at this position. The mutation can disturb this special conformation. The mutation is located in a region with known splice variants, described as *p* > *L* (in dbSNP: rs676210) [43]. The rs1042034 variant also was analyzed in a Framingham cohort and its descendant generation; in contrast to our results, they found that C/C genotype increases the risk of premature onset of cardiovascular diseases mainly in men (RR = 2.18, *p* = 4.5 × 10^−5^) [44]; however, we found in our study population that the rs1042034 variant reduces the likelihood of having an ACS (OR = 0.05, *p*=0.037), and it should be noted that we found a marked difference in the presence of homozygotes for the T/T genotype between groups (ACS 5% vs. 9.7% CG), and the wild allele C is more frequent in the ACS group (25% ACS vs. 28.2% CG); this is the first time that this variant has been studied in our population. This variant confers a change (Ser > Asn) in the position 4338 of protein [45], and in the *α*3 domain, this domain has reversible lipid affinity [46–48], asparagine residue is bigger and less hydrophobic than serine. Hydrophobic interactions, either in the core of the protein or on the surface, will be lost. The wild-type residue is very conserved, but a few other residue types have been observed at this position too. The mutant residue was not among the other residue types observed at this position in other, homologous proteins. However, residues that have some properties in common with the mutated residue were observed. This means that in some rare cases, the mutation might occur without damaging the protein [43].

We analyze APOB levels according to the dominant and recessive inheritance models; in this analysis, we only found an association in the control group for the recessive model with the rs1469513 variant (*p* < 0.0001); therefore, carriers of two C alleles could have higher levels of APOB, and it could be expected that in the ACS group, the contribution of this allele to have higher levels of APOB is masked by lipid-lowering drugs. This finding has been observed for the first time in our population and has not been analyzed in any other.

We found linkage disequilibrium between the pair variants (*p* < 0.05), and we found that the haplotype TAGT confers a risk 2.14 times higher (*p* < 0.001).

Variant rs1469513 (T > C) has not been reported in linkage disequilibrium in previous studies; in this study, we found a high linkage disequilibrium with rs676210 (*D*′ = 0.83, *p*=0.0001, *r*^2^ = 0.15, *p*=0.0001).

Variants rs676210 and rs1042034 have been found in linkage disequilibrium previously. A study reported that the haplotypes ATGGA and ATAGG formed by rs1042034, rs2163204, rs512535, rs676210, and rs679899 are a risk for hyperlipidemia (OR: 1.46, *p*=0.04, OR: 1.63, *p*=0.04) [10].

In another study, the haplotype TGAG (rs1042034, rs676210, rs693, and rs673548 was associated with an increased risk of ischemic stroke (OR = 1.583, *p*=0.031) [49].

Our findings demonstrate the impact that the genetic variants studied have on the risk of ACS in our population, which opens the possibility of conducting studies where their role in the response to drugs is analyzed, which would allow developing strategies for medicine personalized. As a limitation of our study, it is difficult to interpret the impact of the variants on the serum levels of APOB since the population that makes up the group of cases consumes drugs that can modify lipids and apolipoprotein levels, and longitudinal studies in individuals without medication could show the impact of genetic variants alone or the haplotype constructed with them on serum levels of APOB in individuals without medication.

In summary, our findings show that the risk of presenting an ACS event in the study population can be modified by the presence of the variants rs673548, rs676210, and rs1042034 and with TAGT haplotype.

## 5. Conclusions

Our findings support a significant risk association between rs673548 and rs676210 variants for ACS; meanwhile, rs1042034 could be considered protective factor in a western Mexican population. Also, in this population, haplotype TAGT may confer 2.14 times a higher risk.

## Figures and Tables

**Table 1:** Clinical and demographic characteristics.

Demographic data	CG *n* (%)	ACSG *n* (%)	*p*
Age (mean)	57.76	64.25	<0.0001
Female	130 (43)	71 (24)	<0.0001
Male	170 (57)	229 (76)	<0.0001
UA	—	31 (10)	—
NSTEMI	—	19.7 (30)	—
STEMI	—	210 (70)	—-
Reinfarction	—	51 (17)	—
Risk factor			
High blood pressure	104 (34.7)	210 (70)	<0.001
Dyslipidemia	61 (20.3)	136 (45.6)	<0.001
T2DM	68 (22.7)	160 (53.3)	<0.001
Smoking	62 (20.7)	144 (48.3)	<0.001
Overweight (kg/m^2^)	71 (23.7)	112 (37.3)	0.003
Obesity (kg/m^2^)	49 (16.3)	92 (30.7)	<0.001
Sedentary lifestyle	69 (23)	165 (55.4)	<0.001
Drug consumption			
Antilipemic	—	266 (89)	—

CG (control group), ACSG (acute coronary syndrome group), T2DM2 (type 2 diabetes mellitus). *p* value of the Mann–Whitney test.

**Table 2:** Biochemical parameters.

Para-clinical	CG Mean (IQR)	ACSG Mean (IQR)	*p*	Reference value
Glucose	125.4 (86–124)	144.15 (99.75–181.5)	0.010	75–105 mg/dL
Cholesterol	169.59 (141–199)	118.64 (92–140)	<0.001	150–199 mg/dL
Triglycerides	123.1 (82–144)	91.74 (72–107)	<0.001	≤250 mg/dL
HDL	41.6 (26–55)	28.4 (13–27)	<0.001	≥40 mg/dL
LDL	77.9 (53–96)	43.5 (33–51.25)	<0.001	≤130 mg/dL
APOB	165.54 (146.7–184.8)	128.4 (103.8–154.4)	<0.001	94–178 mg/dL
CRP	4.2 (1–4)	26.18 (5.05–37)	<0.001	1–10 mg/L
Troponine I	—	544.24 (0.6–9.8)	—	0.1–4 ng/dL
CK	—	838.7 (149–951)	—	24–195 (U/L)
CK-MB	—	109.8 (23–125)	—	≤130 (U/L)

CG (control group), ACSG (acute coronary syndrome group), IQR (interquartile range), HDL (high-density lipoproteins), LDL (low-density lipoproteins), APOB (apolipoprotein B), CRP (C-reactive protein), CK (creatine kinase), CK-MB (creatine kinase MB). *p* value of the Mann–Whitney test.

**Table 3:** Genotype and allele distribution of the rs1469513, rs673548, rs676210, and rs1042034 in the APOB gene in CG and ACSG.

	CG *n* (%)	ACSG *n* (%)	OR CI (95%)	*p*
rs1469513				
Genotype				
T/T	99 (33)	113 (37.7)	—	—
T/C	143 (47.7)	143 (47.7)	0.87 (0.61–1.25)	0.466
C/C	58 (19.3)	44 (14.6)	0.66 (0.41–1.07)	0.090
Allele				
T	341 (58.8)	369 (61.5)	—	—
C	259 (43.2)	231 (38.5)	0.82 (0.65–1.03)	0.100
rs673548				
Genotype				
G/G	181 (60.3)	151 (50.3)	—	—
G/A	98 (32.7)	125 (41.7)	1.53 (1.08–2.15)	0.014
A/A	21 (7)	24 (8)	1.37 (0.734–2.55)	0.321
Allele				
G	460 (76.66)	427 (71.16)	—	—
A	140 (23.33)	173 (28.8)	1.33 (1.02–1.72)	0.030
rs676210				
Genotype				
G/G	185 (61.7)	141 (47)	—	—
G/A	95 (31.7)	118 (39.3)	1.63 (1.15–2.31)	0.005
A/A	20 (6.6)	41 (13.7)	2.69 (1.50–4.79)	<0.001
Allele				
G	465 (77.5)	400 (66.7)	—	—
A	135 (22.5)	200 (33.3)	1.72 (1.333–2.225)	<0.001
rs1042034				
Genotype				
C/C	160 (53.3)	165 (55)	—	—
T/C	111 (37)	120 (40)	1.05 (0.75–1.47)	0.784
T/T	29 (9.7)	15 (5)	0.50 (0.26–0.97)	0.037
Allele				
C	431 (71.8)	450 (75)	—	—
T	169 (28.2)	150 (25)	0.85 (0.66–1.09)	0.214

CG (control group), ACSG (acute coronary syndrome group), OR (odds ratio), CI (confidence interval).

**Table 4:** Differences between groups of the rs1469513, rs673548, rs676210, and rs1042034 by the inheritance model.

	CG *n* (%)	ACSG *n* (%)	OR CI (95%)	*p*
rs1469513				
Dominant model				
T/T	99 (33)	113 (37.7)	—	—
T/C + C/C	201 (67)	187 (62.3)	0.81 (0.58–1.14)	0.231
Recessive model				
T/T + T/C	242 (80.7)	256 (85.4)		
C/C	58 (19.3)	44 (14.6)	0.71 (0.46–1.10)	0.128
rs673548				
Dominant model				
G/G	181 (60.3)	151 (50.3)	—	—
G/A + A/A	119 (39.7)	149 (49.7)	1.50 (1.08–2.07)	**0.013**
Recessive model				
G/G + G/A	279 (93)	276 (92)	—	
A/A	21 (7)	24 (8)	1.15 (0.62–2.12)	0.641
rs676210				
Dominant model				
G/G	185 (61.7)	141 (47)	—	—
G/A + A/A	115 (38.3)	159 (53)	1.81 (1.31–2.51)	**<0.001**
Recessive model				
G/G + G/A	280 (93.4)	259 (86.3)	—	—
A/A	20 (6.6)	41 (13.7)	2.21 (1.265–3.88)	**0.004**
rs1042034				
Dominant model				
C/C	160 (53.3)	165 (55)	—	—
T/C + T/T	140 (46.7)	135 (45)	0.93 (0.67–1.28)	0.682
Recessive model				
C/C + T/C	271 (90.3)	285 (95)	—	—
T/T	29 (9.7)	15 (5)	0.49 (0.25–0.93)	**0.028**

CG (control group), ACSG (acute coronary syndrome group), OR (odds ratio), CI (confidence interval).

**Table 5:** Haplotype analysis of the rs1469513, rs673548, rs676210, and rs1042034 in the APOB gene in CG and ACSG.

Haplotype	CG *n* (%)	ACSG *n* (%)	OR CI (95%)	*p*
CGAC	212 (35.4)	172 (28.6)	—	—
TGAC	174 (28.9)	188 (31.3)	1.33 (0.99–1.77)	0.051
TAGT	72 (12.1)	125 (20.8)	2.14 (1.50–3.04)	**<0.001**

CG (control group), ACSG (acute coronary syndrome group), CI (confidence interval), OR (odds ratio). Haplotype with frequency lower than 5% is not shown. Haplotype is represented by rs1469513, rs673548, rs676210, and rs1042034.

**Table 6:** APOB levels in ACSG adjusted by independent variables.

	Beta coefficient	Confidence interval		*p* value
		Lower limit	Upper limit	
Constant	33.245	1.506	64.985	0.040
Age	0.178	−0.175	0.531	0.321
Sex	3.765	−4.266	11.796	0.356
BMI	0.332	−3.862	4.525	0.876
Diabetes	−1.438	−9.880	7.005	0.737
Hyperlipidemia	0.494	−6.076	7.063	0.882
HBP	−3.116	−10.330	4.097	0.395
Smoking	−2.233	−8.725	4.259	0.498
Physical activity	2.357	−4.276	8.990	0.484
Cholesterol	**0.779**	**0.633**	**0.926**	≤**0.001**
Glucose	0.052	−0.012	0.116	0.109
Triglycerides	−0.081	−0.215	0.054	0.238
HDL	**−0.597**	**−0.935**	**−0.258**	**0.001**
LDL	0.198	−0.006	0.402	0.057
Statins	0.612	−12.275	13.500	0.925
Fibrates	22.281	−25.668	70.230	0.361

Dependent variable: APOB levels. Abbreviations: BMI (body mass index), HBP (high blood pressure), HDL (high-density lipoprotein), LDL (low-density lipoprotein).

## Data Availability

The datasets generated during the present study are not publicly available because they are cited in the text.
